# Advent of Artificial Intelligence in Spine Research: An Updated Perspective

**DOI:** 10.3390/jcm15020820

**Published:** 2026-01-20

**Authors:** Apratim Maity, Ethan D. L. Brown, Ryan A. McCann, Aryaa Karkare, Emily A. Orsino, Shaila D. Ghanekar, Barnabas Obeng-Gyasi, Sheng-fu Larry Lo, Daniel M. Sciubba, Aladine A. Elsamadicy

**Affiliations:** 1 Department of Neurosurgery, Donald and Barbara Zucker Hofstra School of Medicine at Northwell, 300 Community Drive, Manhasset, NY 11030, USA; amaity@northwell.edu (A.M.); ebrown35@northwell.edu (E.D.L.B.); rmccann1@northwell.edu (R.A.M.); akarkare@northwell.edu (A.K.); eorsino@northwell.edu (E.A.O.); larrylo@northwell.edu (S.-f.L.L.); dsciubba1@northwell.edu (D.M.S.); 2 Yale School of Medicine, Yale University, New Haven, CT 06510, USA; shaila.ghanekar@yale.edu; 3 Department of Neurosurgery, Allegheny Health Network, Pittsburgh, PA 15212, USA; barnabas@ahn.org

**Keywords:** artificial intelligence, machine learning, spine surgery, deep learning, imaging analysis, predictive modeling, clustering, phenotype discovery, natural language processing, large language models, hybrid modeling, generalizability, clinical decision support

## Abstract

Artificial intelligence (AI) has rapidly evolved from an experimental tool in spine research to a multi-domain framework that has significantly influenced imaging analysis, surgical decision-making, and individualized outcome prediction. Recent advances have expanded beyond isolated applications, enabling automated image interpretation, patient-specific risk stratification, discovery of qualitative phenotypes, and integration of heterogeneous clinical and biomechanical data. These developments signal a shift toward more comprehensive, context-aware analytic systems capable of supporting complex clinical workflows in spine care. Despite these gains, widespread clinical adoption remains limited. High internal performance metrics do not consistently translate into reliable generalizability, interpretability, or real-world clinical readiness. Persistent challenges, which include dataset heterogeneity, transportability across institutions, alignment with clinical decision-making processes, and appropriate validation strategies, continue to constrain widespread implementation. In this perspective, we synthesize post-2019 advances in spine AI across key application domains: imaging analysis, predictive modeling and decision support, qualitative phenotyping, and emerging hybrid and language-based frameworks through a unified clinical-readiness lens. By examining how methodological progress aligns with clinical context, validation rigor, and interpretability, we highlight both the transformative potential of AI in spine research and the critical steps required for responsible, effective integration into routine clinical practice.

## 1. Introduction

Artificial intelligence (AI), within which machine learning (ML) represents a central methodological paradigm, has rapidly moved from theoretical promise to practical experimentation across nearly every domain of medicine, and spine care has been no exception [1]. The spine is uniquely suited to AI-driven analysis: it generates rich imaging, biomechanical, and clinical datasets, yet decision-making often remains subjective and highly variable across providers and institutions. In 2019, Galbusera et al. published the most recent major review of AI in spine research, capturing a moment when classical machine learning approaches dominated, deep learning was just beginning to enter imaging pipelines, and questions of data governance, external validity, and ethical deployment were only starting to surface [1]. During this time, support vector machines (SVM), decision trees, and random forests (RF) were commonly used for tasks such as vertebral localization, scoliosis classification, and early outcome prediction [1]. Deep learning architectures, particularly convolutional neural networks, were only beginning to emerge within imaging pipelines [1]. Although early studies demonstrated that AI models could match expert-level performance in narrowly defined tasks, limitations related to data scarcity, lack of external validation, computational demands, interpretability, and ethical governance constrained widespread clinical translation [1].

Five years later, the field looks markedly different. Deep learning (DL) models hold a majority stake in the automation of image analysis, while predictive algorithms help inform risk stratification and surgical planning. Clustering techniques, which rely on unsupervised machine learning, are able to reveal qualitative phenotypes that reshape how we conceptualize disease. Hybrid modeling, natural language processing (NLP), and large language models (LLM) have shown the ability to integrate previously unexplored data streams [2,3,4]. Yet despite these advances, issues still remain regarding the generalizability and interpretability of these models, further hindering their widespread implementation in the clinical world. As such, continuously monitoring the status of AI in spine research is crucial as we explore new frontiers for the application of AI within medicine.

To construct this perspective, we performed a comprehensive PubMed search to identify English-language, human-subject studies describing applications of artificial intelligence in spine research published from 2020 onward. In our search strategies, we used a combination of MeSH terms and text-word queries that spanned both artificial intelligence in general, as well as machine learning methods in particular, with a focus on spine-related conditions and procedures. Retrieved records underwent an initial title and abstract screen to exclude clearly irrelevant studies and secondary literature, followed by full-text review of eligible articles to characterize the specific clinical or research problem addressed, the data modality utilized (e.g., imaging, clinical registries, unstructured text, etc.), and the primary analytic objective. Using this approach, we identified recurring application domains of post-2019 work that represented the most active and clinically relevant areas of advancement. In order to maintain a unified perspective rather than a series of parallel topic summaries, we organize post-2019 advances around these domains where AI has most directly altered clinical workflows in spine care: imaging analysis, risk stratification/decision support, qualitative phenotyping, and emerging hybrid/NLP/LLM paradigms. Across each domain, we interpret progress through a consistent clinical-readiness lens that includes discussion surrounding (i) data representativeness and heterogeneity, (ii) validation and transportability, (iii) interpretability and accountability, and (iv) implementation feasibility. At the end of the perspective, we synthesize these considerations through a reflective comparison with the 2019 vision, evaluating the extent to which post-2019 advances have translated into clinically meaningful readiness and clarifying the remaining methodological and contextual barriers to implementation.

## 2. Understanding Artificial Intelligence: Math, Models, and More

**Figure 1:**
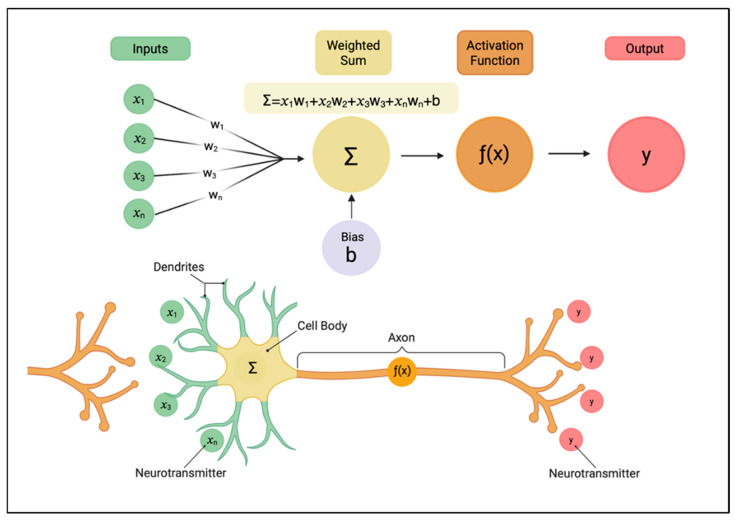
Conceptual and mathematical structure of a perceptron within a connectionist model. A perceptron is the fundamental computational unit of a connectionist model, which is shown above in both its biological and mathematical forms. At the top, the perceptron receives a set of input features, x1, x2, …, xn, which are each scaled by an associated weight w1, w2, …, wn. These weighted inputs are summed together with a bias term b to produce a weighted sum which is then passed through an activation function, f(x), that introduces nonlinearity. This generates the final output y. The lower panel depicts an analogous biological neuron, where dendrites collect incoming neurotransmitter signals, the cell body integrates them, and the axon transmits an output signal. This analogy showcases why perceptrons are often described as simplified mathematical abstractions of real neurons [9].

**Figure 2:**
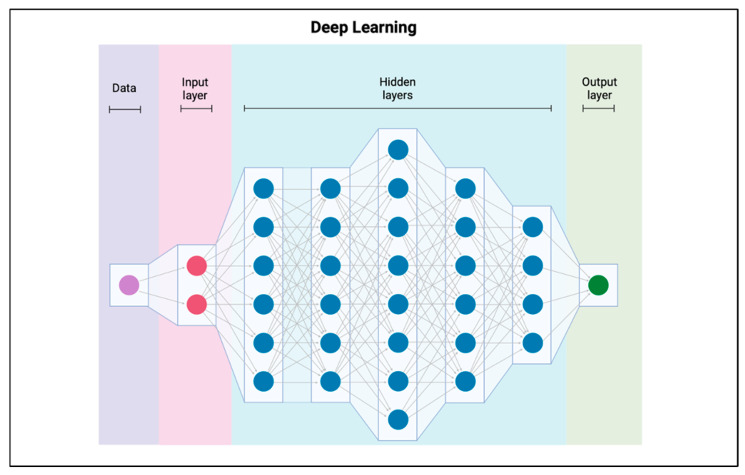
Architecture of a deep learning neural network. Deep learning represents a class of machine learning models within artificial intelligence in which input data are processed through multiple interconnected layers. Each successive layer extracts increasingly abstract features from the data, allowing the model to learn complex patterns and relationships and ultimately generate a prediction.

Artificial intelligence is a field of computer science dedicated to creating systems capable of performing tasks that normally require human intelligence, such as perceiving, reasoning, learning, and decision-making [5]. It is an umbrella term that includes various approaches, from simple rule-based reasoning to complex neural networks [5]. A core approach within AI is machine learning, which is defined as the way a system is taught intelligence through the feeding of training data. Training data allows the model to discover patterns on its own, and in this way, the model learns from “experience” rather than explicit programming [6]. Traditional ML techniques often rely heavily on humans to select and extract specific features (e.g., measuring disc height) before feeding them into the model [7]. In contrast, deep learning is a sophisticated type of ML architecture that uses multi-level “deep” networks to automatically learn progressively more abstract representations of raw data [8]. See Figure 1 for an overview of how a perceptron, the fundamental unit of a DL network works. See Figure 2 for an overview of a DL architecture. The engine behind deep learning is the artificial neural network (ANN), a connectionist model mathematically inspired by the structural network of the human brain (Figure 1 and Figure 2) [8]. An ANN consists of multiple layers of interconnected processing units called perceptrons. 

Machine learning algorithms are primarily categorized into five main forms based on how they learn: supervised, unsupervised, semi-supervised, self-supervised, and reinforcement learning. Supplementary Table S1 summarizes each type of ML algorithm and provides examples of their applications within spine research. The models themselves represent unique architectures that learn using these various ML algorithms. Each model is often well-suited for a particular application, depending on the operational framework that optimally analyzes the respective dataset. Supplementary Table S2 provides a detailed overview of the most commonly used model types in spine research, summarizing their learning mechanisms and how each model operates in practice.

While understanding these core architectures is fundamental, equally important is grasping how AI models are trained, validated, and evaluated. During training, a dataset is typically divided into distinct subsets: the training set, which represents the data the model learns from; the test set, reserved for final evaluation on unseen data; and often a validation set, used to fine-tune parameters and prevent overfitting. Overfitting occurs when a model performs exceptionally well on its training data but fails to generalize to data that it hasn’t seen before. To measure model performance objectively, researchers employ various quantitative metrics tailored to the task. Supplementary Table S3 summarizes commonly reported performance metrics in AI and ML research.

As AI systems have grown in complexity, the question of “interpretability” has also become central to their responsible use [10]. Traditional algorithms such as linear regression or decision trees are inherently interpretable because their decision logic can be traced explicitly. In contrast, deep learning and ensemble models often function as “black boxes,” producing highly accurate outputs without transparent reasoning [11,12]. To address this limitation, methods such as saliency mapping, feature-importance ranking, and rule-extraction frameworks have been developed to visualize or approximate how input features influence model predictions. Interpretability directly supports ethical AI by fostering clinician trust, mitigating bias, and ensuring accountability in automated decision-making.

## 3. Advances in Imaging Analysis

Since 2019, the domain of imaging analysis has undergone rapid maturation due to the introduction of sophisticated LLMs like ChatGPT [13,14]. Such imaging analysis has evolved from basic segmentation to advanced, clinically focused applications. This progress is unsurprising, however, as “Radiology and Medical Imaging” represents the most active research area for deep learning applications within the spine [12].

Early post-2019 efforts were primarily aimed at conquering foundational imaging tasks such as localization, segmentation, and automated quantification [12]. In a cross-sectional study that utilized a training and test dataset, each consisting of 50 T2-weighted sagittal lumbar spine MRIs, Huang et al. developed and evaluated a DL-based program called Spine Explorer for the automated segmentation and quantification of vertebrae and intervertebral discs [15]. Huang et al. found that the trained Spine Explorer automatically segments and measures a lumbar MRI in half a second, achieving a mean Intersection-over-Union (IoU) value of 94.7% for vertebral segmentation and 92.6% for disc segmentation in the test dataset [15]. Furthermore, the agreements for measurements acquired using automated Spine Explorer and manual ImageJ were excellent for both vertebrae and disc measurements (intraclass correlation coefficient = 0.81–1.00) [15].

This type of AI segmentation performance extends beyond just the vertebrae and discs. In a cross-sectional observational study consisting of 120 T2-weighted axial MRIs taken at the L4–5 disc level, Shen et al. adapted the Mask R-CNN framework to create a DL-based program called Spine Explorer (Tulong) for the automated segmentation and quantification of major lumbar spine components, focusing specifically on the paraspinal muscles [16]. Shen et al. reported that Spine Explorer (Tulong) also measures an axial lumbar MRI in 1 s, achieving IoU segmentation values ranging from 83.3% to 88.4% for the paraspinal muscles [16]. Furthermore, when comparing measurements acquired by Spine Explorer (Tulong) with those manually acquired by ImageJ (version 1.80), the inter-software agreements were excellent (ICC = 0.85–0.99) for various size and compositional measurements of the bilateral multifidus, erector spinae, and psoas muscles [16].

Building upon these foundations, AI has made significant strides in computer-aided diagnosis and classification. In 2017, Jamaludin et al. introduced and evaluated SpineNet, a CNN capable of performing automated classification and evidence visualization to read radiological features of the lumbar spine, with a focus on Pfirrmann grading of disc degeneration [17]. In their multicenter retrospective study, which utilized lumbar MR imaging of 12,018 intervertebral discs from 2009 patients, Jamaludin et al. found that the automated SpineNet system achieved an agreement of 70.1% with expert radiologists, which was very similar to the reported inter-rater agreement between those same distinct expert radiologists of 70.4% [17]. This demonstrated that the automated system could perform the reading of radiological features from MRIs without human intervention and achieve diagnostic accuracy equivalent to that of a trained radiologist. Three years later, in 2020, Ishimoto et al. conducted a validation study on the SpineNet system utilizing MRI scans from 971 participants taken from the large population-based Wakayama Spine Study cohort [18]. They, however, trained the SpineNet system to grade central Lumbar Spinal Stenosis (LSS) qualitatively into four grades (“none”, “mild”, “moderate”, and “severe”) [18]. The authors found that the overall agreement rate between the automated grading and the reference standard for all four grades was 65.7% with a reliability (Lin’s correlation coefficient) of 0.73 [18]. Furthermore, when LSS was classified dichotomously into two groups (“severe” vs. “no/mild/moderate”), the system achieved an agreement rate of 94.1% with a kappa of 0.75 [18]. With this strong display of generalizability, the SpineNet system displayed its foundational utility for delivering the reproducible metrics that are required for large-scale epidemiological studies of complex spinal disorders.

Disc degeneration and LSS are not the only spinal pathologies whose diagnostic classification has been increasingly informed by AI-driven analytical frameworks in recent years. The application of hybrid DL models in the assessment of subtle, clinically complex pathologies such as Modic changes (MCs) has also seen strong performance. In a retrospective analysis of 140 patients with MCs who underwent MRI diagnosis, Liu et al. developed and evaluated an intelligent assisted diagnosis system that utilized a Single Shot Multibox Detector network for lesion localization and a ResNet18 network for lesion classification to automatically detect and classify the MCs [19]. Liu et al. found that the hybrid model achieved an accuracy of 86.25% for the detection and classification of MCs, which resulted in an observer-classifier diagnosis consistency kappa value of 0.717 [19]. These results show that AI performance is now not only achieving similar consistencies with clinicians in the assessment of more obvious spinal pathologies, but also in the evaluation of more subtle and complex ones.

Beyond MRI-based evaluation, DL has also been successfully applied to the analysis of CT imaging. In a retrospective study utilizing 452 CT scans of the lumbar/thoracolumbar spine, Glessgen et al. introduced and evaluated a DL pipeline that was trained for the detection of opportunistic fractures in routine CT spine images of varying fields-of-view [20]. Glessgen et al. found that when applied to the 330 correctly segmented vertebrae in the test set, the classifier detected fractures with 88% sensitivity and 95% specificity, achieving an overall accuracy of 93% [20]. DL frameworks have also begun to support the objective assessment of curvature and alignment on X-ray as well [21]. In a retrospective study that utilized both the MICCAI 2019 AASCE Challenge Dataset (98 images for testing) and the BUU LSPINE Dataset (80 images for testing), Xiang et al. introduced and evaluated the Vertebrae Localization and Detection Network (VLD-Net), a model that utilizes a deep reinforcement learning architecture to define vertebrae localization [21]. Xiang et al. found that VLD-Net achieved an average inference time of 0.14 s per X-ray image and significantly improved the precision of scoliosis assessment by achieving a mean absolute error of the Cobb angle within 3° for angles ranging from 0° to 40°, and 4.31° for angles exceeding 40° (severe scoliosis cases) [21]. These errors fall within the acceptable range for clinical applications, as Cobb angle recognition errors within 5 degrees are usually tolerated [21].

The latest frontier in spine imaging involves multimodal integration and synthetic image generation, aimed at overcoming the persistent limitations of data heterogeneity and acquisition time [22,23]. In a comprehensive experimental study utilizing three datasets, including a hip dataset (*n* = 57 patients), a lumbar spinal dataset (*n* = 17 patients), and a thoracic spinal dataset (*n* = 55 patients), Gao et al. proposed and evaluated a novel weakly supervised anatomy-aware multimodal articulated image registration (MAIR) network, referred to as MAIRNet [23]. The MAIRNet architecture was designed to solve the hybrid rigid-elastic registration problem, where, in order to achieve proper registration of the spine, a model needs to maintain rigidity for bony structures while allowing elastic deformation for surrounding soft tissues. Gao et al. found that MAIRNet achieved superior performance over state-of-the-art methods in registration accuracy, with a DSC of 90.8% for the pelvis on the hip dataset and 86.1% for the L4 vertebra on the lumbar spinal dataset [23]. Their model demonstrated better bony structure rigidity preservation, while only requiring the segmentation masks of bony structures in the fixed CT data for supervision [23].

DL algorithms can also now synthesize high-quality STIR MR images from conventional T1- and T2-weighted scans, and testing has shown these synthetic images to be diagnostically interchangeable with conventionally acquired STIR images [22]. In a multicenter, multireader trial consisting of 110 spine MR imaging studies from 93 patients, Tanenbaum et al. used a CNN-based software (SubtleSYNTH) to generate synthetic STIR (Syn-STIR) MR images from acquired sagittal T1- and T2-weighted images [22]. Amazingly, Tanenbaum et al. concluded that the Syn-STIR images were diagnostically interchangeable with conventionally acquired STIR (Acq-STIR) [22]. Not only were they diagnostically interchangeable, but the Syn-STIR images also demonstrated significantly higher image quality (mean paired difference = 0.50; CI: 0.33–0.67; *p* < 0.0001) [22]. Furthermore, in a separate analysis conducted by the same group where five radiologists evaluated the presence or absence of prevertebral fluid collections, bone edema, or posterior soft-tissue/ligamentous injury on STIR imaging in patients with trauma, Tanenbaum et al. found that interchanging Syn-STIR in place of Acq-STIR images resulted in an overall increase in interreader agreement by 11.9% across the three classified injury classes [22].

Together, these advances mark a pivotal evolution in the role of AI within spine imaging, with the field progressing from passive analysis of existing data toward active enhancement and synthesis of clinically actionable imaging information. At the same time, the imaging domain illustrates several cross-cutting challenges that recur throughout contemporary spine AI research. High internal performance metrics and diagnostic parity with expert readers do not consistently translate into reliable performance across institutions, scanners, or acquisition protocols [19]. This fact underscores persistent limitations in generalizability and transportability of these models, and as imaging models become increasingly complex, issues of validation design, interpretability, and standardization become central determinants of clinical readiness.

## 4. Risk Stratification, Decision Support, and Outcome Prediction

Following the early dominance of imaging applications, predictive modeling emerged as the next major domain of AI in spine research to experience rapid advancement. Before 2020, most outcome prediction studies focused on identifying statistical associations between patient/surgical factors and outcomes like surgical complications, revision surgeries, or hospital readmission [1]. However, these analyses were typically limited to single-center datasets and conventional regression models. As a result, while they provided valuable descriptive insights, they lacked the precision and scalability necessary for individualized forecasting [1].

The value of scores and indices in the management of complex patients should not be understated, however. Patients with spinal metastases are prime examples of such complexity, often requiring clinicians to navigate a rough and unpredictable clinical landscape. Skillful management requires the balancing of systemic disease, surgical risk, and functional goals while ensuring that the patient’s overall hospital course points towards a meaningful outcome. In a cohort study of 1613 patients with spinal metastases, Elsamadicy et al. found that the Metastatic Spinal Tumor Frailty Index (MSTFI) was the most sensitive predictor of nonroutine discharge and readmission, outperforming the Charlson Comorbidity Index (CCI) and Modified 5-Item Frailty Index (mFI-5) in identifying patients at risk for adverse outcomes [24]. In a subsequent analysis of 659 elderly patients, the same group demonstrated that the Geriatric Nutritional Risk Index (GNRI), which is a measure of nutritional status, was a superior predictor of 30-day mortality, extended length of stay, and overall complications compared to traditional frailty measures [25]. Therefore, even in the absence of AI and ML, traditional statistical scores and indices possess tremendous clinical utility.

Interestingly, these established statistical indices helped form the scaffolding upon which AI and ML build [11,12,26]. Incorporating statistical scores as model features within machine-learning pipelines has proven valuable for improving outcome prediction. In a retrospective cohort study of 4346 adult patients who underwent surgical intervention for metastatic spinal column tumors, Elsamadicy et al. used an RF model to construct predictive models for 30-day unplanned readmission (AUC = 0.60) and found that the Hospital Frailty Risk Score (HFRS) was the most important feature in predicting readmission risk [27]. Of note, within their study, “intermediate frailty” (HFRS = 5–14.9) was found to be an independent predictor of unplanned 30-day readmission (OR: 1.32; CI: 1.06–1.64; *p* = 0.012) [27]. As innovation advanced, the modeling of complex, nonlinear relationships among clinical, radiographic, and perioperative variables allowed algorithms such as RFs, gradient boosting trees, and neural networks to exceed the performance of traditional statistical techniques [11,26,28,29,30,31]. Early examples demonstrated that ensemble learning could replicate operative decision-making in adult spinal deformity (ASD) with good accuracy, proving that AI could anticipate surgical strategy rather than merely describe correlations [29]. For example, in a retrospective cohort study of 1503 patients with ASD, Durand et al. found that their RF model achieved 79% accuracy in discriminating between patients who required operative versus nonoperative management [29].

Building upon these foundations, more recent models have expanded beyond replicating surgeon behavior towards true prognostic capability [11,28,31,32]. In other words, these models forecast not just what surgeons may do, but what functional outcomes patients may experience. In a cohort study using a nationwide registry of over 2700 patients undergoing anterior cervical discectomy and fusion, Buwaider et al. evaluated multiple supervised-learning frameworks, including logistic regression, gradient boosting trees, k-nearest neighbors, and RFs, to predict persistent postoperative dysphonia [28]. Among these, the RF model achieved the highest discrimination (AUC = 0.79; sensitivity > 0.85) and identified neck disability index, EQ-5D health-status score, and number of fused vertebrae as key predictors [28]. The prediction of mechanical complications has also been tested. In a cohort study of 295 adult spinal deformity patients using pre-, intra-, and postoperative variables to predict mechanical complications, Balaban et al. applied an RF model, which achieved 72% accuracy and 91% sensitivity for detecting events such as junctional failure, rod breakage, and implant-related complications [11]. These studies helped shift the view of events deemed largely unpredictable prior to 2019 towards possibly quantifiable with clinically meaningful precision.

In conjunction with the aforementioned functional outcomes, ML-based risk stratification has also expanded to encompass quality-of-life outcomes. In a multicenter retrospective study of 848 patients undergoing lumbar decompression for spinal canal stenosis, Yagi et al. used an ensemble model to predict 2-year postoperative quality-of-life outcomes and achieved strong correlations (r = 0.82–0.89) between predicted and observed results [30]. In a separate multicenter study of 554 patients with degenerative cervical myelopathy, Shakil et al. used LASSO regression and XGBoost models with 14 preoperative variables to predict 12-month quality-of-life improvement, achieving good accuracy (AUC = 0.75 and 0.74, respectively, for Neck Disability Index) and identifying both shorter symptom duration and worse baseline status as key predictors of treatment response [31]. The successful application of ML in predicting these detailed, patient-reported outcomes not only moves spine care beyond generalized estimates but also lays the foundation for truly individualized risk stratification.

Importantly, these predictive advances are now being operationalized into clinical decision-support tools [11,33]. Prior to the advent of validated ML calculators, surgical risk estimation for complex spine procedures was often highly discordant and heavily reliant on individual surgeon intuition [33]. In an observational study of 39 experienced spine surgeons from 12 countries, Pellisé et al. found striking variability in risk estimates for adult spinal deformity cases ranging from 1% to 100% for identical scenarios with poor agreement among surgeons (intraclass correlation coefficient = 0.10–0.32) [33]. Contemporary ML-based risk calculators would address this gap by providing consistent, objective risk estimates that can guide preoperative counseling and enhance shared decision-making.

Finally, as predictive systems grow more complex, interpretability has become a central limitation that has stalled widespread clinical adoption. Frameworks such as interpretable trees (inTrees) applied to RF models and gradient boosted trees have enabled researchers to extract concise, rule-based explanations that help clarify model logic [11]. This focus on transparency and usability represents a maturing trajectory of AI in spine research: from descriptive statistics and opaque algorithms to interpretable, clinically embedded tools capable of delivering personalized, evidence-based forecasts for patients across the full spectrum of spinal pathology.

Collectively, advances in predictive modeling and decision support illustrate both the promise and the translational challenges of AI in spine care. While contemporary models have increasingly achieved strong discrimination for complications, functional outcomes, and quality-of-life metrics, their clinical value ultimately depends on factors that extend beyond performance alone. Model generalizability across institutions, calibration to evolving practice patterns, interpretability of predictions, and alignment with real-world clinical decision-making workflows remain critical determinants of widespread adoption. As such, even technically mature risk stratification tools must be evaluated and deployed within their intended clinical context, because failure to do so risks overreliance on predictions that may not be generalizable, interpretable, or aligned with real-world decision-making.

## 5. Qualitative Phenotyping

Efforts to uncover distinct subtypes within complex spine conditions have led researchers to explore unsupervised machine learning, particularly clustering, for qualitative phenotyping [2]. Unlike traditional methods that rely on expert-defined single metric thresholds, such as a specific Cobb angle cutoff or sagittal vertical axis measurement, clustering utilizes comprehensive baseline patient data to discern natural, inherent data subgroups [2,34]. Such patient data may include demographics, patient-reported outcomes (PROs), and frailty indices. This approach has been widely adopted across a diverse array of spinal disorders, from ASD to pediatric scoliosis, aiming to move classification beyond purely anatomical description and towards predicting individualized patient trajectories [2,34].

In ASD, where patient complexity is high due to age and comorbidities, clustering analysis has yielded crucial, non-radiographic patient classifications [2,34]. In a multicenter prospective cohort study of 563 ASD patients, Mohanty et al. applied k-means clustering based on baseline PROs, Edmonton frailty, age, surgical history, and overall health to discern four distinct preoperative phenotypes: “Old/Frail/Content” (OFC) (27.7%), “Old/Frail/Distressed” (OFD) (33.2%), “Old/Resilient/Content” (ORC) (27.2%), and “Young/Resilient/Content” (YRC) (11.9%) [2,34]. Mohanty et al. concluded that the patient’s preoperative qualitative phenotype primarily determines their ability to improve their PROs following surgery and demonstrated that the OFD cluster, which were the worst in terms of preoperative state, improved the least in PROs and had significantly worse two-year reoperation outcomes compared to the OFC cohort (HR: 3.303; CI: 1.085–8.390; *p* < 0.05) [2,34]. Such data-driven classification, focused on function and mental health rather than just radiographic indices, is essential for refining patient counseling and individualized care planning.

The utility of unsupervised phenotyping is equally transformative in pediatric spine care, addressing limitations in long-standing, expert-defined systems. For early onset scoliosis (EOS), the conventional “Classification of EOS” organizes patients into 48 subgroups, but this classification is based on static cutoff values and lacks a clear data-driven foundation to guide surgical consensus or correlate interventions with outcomes [34]. In a retrospective cohort study of 1114 EOS patients across four etiologies (congenital, idiopathic, neuromuscular, and syndromic), Viraraghavan et al. employed the Fuzzy C-means clustering algorithm to create an automated clustering technique based solely on preoperative clinical indices and successfully identified three unique, data-driven subgroups per etiology [34]. Viraraghavan et al. concluded that the adoption of this automated clustering framework can help improve the standardization of clinical decision-making for EOS, as these three clusters per etiology were found to be significantly different from each other for all the clinical indices used (*p* < 0.01) [34]. This objective, data-driven grouping, whether defined by patient function or complex morphological features, offers a far more granular and prognostic view of the disease than simple measurement-based grading.

Collectively, these studies illustrate how qualitative phenotyping represents a conceptual shift from classification systems defined by expert-selected thresholds towards data-driven subgrouping based on the data itself. This domain highlights some important translational considerations, however. The clinical utility of phenotypes depends on their stability across cohorts, reproducibility across institutions, and interpretability for clinicians who must eventually act on these classifications in practice. Without careful validation and contextual grounding, unsupervised clusters risk being statistically distinct yet clinically ambiguous. As such, qualitative phenotyping exemplifies both the potential and the limitations of unsupervised learning in spine care, reinforcing the need to align data-driven discovery with clinical meaning, generalizability, and actionable decision-making.

## 6. Emerging Frontiers: Hybrid Modeling, NLP, and LLMs

Within the past five years, a distinct and more recent evolution within spine research has centered on hybrid AI modeling. This focus reflects the growing need to capture the multifactorial and heterogeneous nature of spinal disorders. Hybrid modeling, as defined in Supplementary Table S2, refers to frameworks that integrate multiple data modalities and complementary AI architectures to model complex biological and biomechanical relationships that single-domain approaches cannot fully capture. In a single-institution analysis of 296 degenerative cervical myelopathy (DCM) patients with community MRI (cMRI) scans and 228 patients with advanced MRI (aMRI) acquisitions, Al-Shawwa et al. compared the accuracy of machine learning models in predicting baseline disease severity between the two imaging protocols [35]. What makes aMRI scans “advanced” is their inclusion of additional MRI techniques, such as diffusion tensor imaging and magnetization transfer sequences. In their study, Al-Shawwa et al. utilized multimodal RF classifiers and found that the model trained on aMRI scans predicted disease severity with 73.3% accuracy, significantly outperforming the model trained on cMRI scans by over 30% [35]. The authors concluded that aMRI metrics perform better in ML models at predicting disease severity of patients with DCM [35].

The MAIR techniques introduced earlier also exemplify this hybrid modeling paradigm. In MAIRNet, the spinal anatomy is modeled according to its biomechanical properties: treating vertebral segments as “rigid” bodies and surrounding soft tissues as “deformable” structures [23]. To handle these differing behaviors, the AI model combines a fixed, rule-based registration step that aligns the rigid bony components with a learnable neural network that adapts to the deformable soft-tissue regions [23]. This design allows MAIRNet to preserve anatomical realism while achieving accurate alignment between CT and MRI modalities [23]. Together, these efforts illustrate how hybrid modeling represents more than just a technical evolution. It marks a conceptual shift toward integrative systems that combine biological insight, imaging precision, and computational inference within unified predictive frameworks.

Parallel to the advances in hybrid modeling is the maturation of NLP. NLP in spine research has been used to leverage AI to extract clinically relevant information, structure, and context from unstructured text across a variety of sources, including electronic health records (EHRs) [4,36,37]. The automation of clinical data acquisition through NLP has helped tackle the costly and labor-intensive process of manual database construction. In a retrospective analysis of 31,502 operative cases retrieved from the NYU Langone Department of Neurosurgery over the last decade, Cheung et al. applied surgeon-written regular expression (regex) classifiers to automatically generate a spine surgery autoregistry from unstructured operative notes within the electronic health record (EHR) [4]. Cheung et al. found that the regex classifiers achieved an overall accuracy of 98.86% for identifying procedures and relevant vertebral levels, with a Cohen kappa statistic of 0.8037, assessing agreement against manually reviewed labels [4]. The authors also demonstrated the system’s utility in monitoring quality metrics by calculating the institutional rate of reoperation within 30 days as 1.97% for all identified spine procedures [4].

Within academic research applications, NLP techniques like topic modeling have been deployed to scrutinize the vast body of academic literature, helping to identify prevalent and emerging research themes [38]. In a study examining 3358 articles and reviews published in The Spine Journal from its inception in 2001 to 2023, Karabacak and Margetis applied the BERTopic NLP technique combined with linear regression models to trace long-term research trends [38]. Their analysis revealed that “Degenerative Cervical Myelopathy”, “Osteoporosis”, and “Opioid Use” were the hottest topics, while “Intradural Lesions”, “Extradural Tumors”, and “Vertebral Augmentation” were the coldest topics [38].

A rather unique application of NLP has been in the analysis of patient-written reviews of spine surgeons. Such analysis has allowed for the quantification of subjective feedback to ultimately provide new insight into patient satisfaction and perception [36]. In a retrospective quantitative analysis of 2239 written and star-rated online reviews of 177 Cervical Spine Research Society (CSRS) surgeons practicing in the United States, Tang et al. applied the Valence Aware Dictionary and sEntiment Reasoner (VADER) NLP package to calculate sentiment scores for each written review [36]. The authors found that words associated with positive surgeon behaviors such as “listens” were positively associated with largely positive reviews (OR: 2.75; CI: 1.27–5.95; *p* = 0.01), while the inclusion of the words “pain” or “severe pain” were significantly associated with decreased odds of receiving positive reviews (OR: 0.34; CI: 0.27–0.43; *p* < 0.01 and OR: 0.16; CI: 0.04–0.76; *p* = 0.02, respectively) [36]. Importantly, the inclusion of the phrase “pain-free” conferred an even greater likelihood of a positive review (OR: 3.69; CI: 1.98–6.90; *p* < 0.01) [35]. These results led the authors to conclude that pain is the most significant factor driving both positive and negative written reviews of CSRS surgeons [36].

The use of NLP in the analysis of patient sentiment extends from formal written reviews to social media posts as well. Such analyses are paramount for understanding the patient experience because they provide unique insights into patient perspectives that may not be captured in traditional clinical settings or formal written reviews. While not directly spine-related, in a social media network analysis of 5466 unique posts across 7 health-related subreddits regarding sarcoma patients, Schneider et al. applied NLP tools to explore how disease status, treatment, and the patient experience interconnect for sarcoma patients [39]. Their study included regex matching for theme detection and VADER sentiment analysis to assess emotional valence [39]. Schneider et al. found that treatment-related discussions dominated the discourse, with chemotherapy and radiation therapy being strongly associated with work-related impacts (OR: 2.85; *p* < 0.001 and OR: 2.47; *p* < 0.001, respectively) [39]. The authors also found that discussion of disease progression both served as a critical transition point in the patient experience and was robustly related to discussion of radiation therapy (OR: 4.09; *p* < 0.001) [39]. Similar large-scale sentiment analyses in spine populations could provide equally valuable insight, as spinal disorders often impose comparable physical, emotional, and functional burdens. Understanding these lived experiences may help surgeons better align treatment strategies with patient expectations and quality-of-life priorities.

Recent advances in LLMs represent the next evolution of NLP, as they enable the generation, summarization, and dissemination of clinical knowledge at an unprecedented scale [40]. This has shown to be especially useful in research studies that explore unstructured text entries within open access databases, such as those exploring the Manufacturer and User Facility Device Experience (MAUDE) database. The challenge of using the MAUDE database is that reports are written as qualitative narratives, and this narrative structure presents methodological difficulties for performing the systematic, large-scale, quantitative analysis that is necessary to quantify things like time impacts or economic implications. LLMs possess the capability to make such research studies significantly more feasible. In a cross-sectional study analyzing 1346 robotic spine surgery adverse events reported to the MAUDE database from 2016 to 2025, Schneider et al. applied the DeepSeek Reasoner API LLM to convert qualitative narrative descriptions into quantifiable variables [41]. Using this method, Schneider et al. were able to show that adverse events resulted in a mean procedural delay of 58.1 min, with precision issues constituting 66.4% of reported complications [41].

LLMs like ChatGPT and Google Bard also hold significant promise for revolutionizing patient education by delivering personalized, easily comprehensible content and facilitating more informed patient-level decision-making [40]. However, it appears that these LLMs haven’t achieved a standard high enough to warrant clinical implementation just yet. In a web review evaluating the utility of LLMs in patient education, Lang et al. sourced 10 critical questions based on frequently asked questions (FAQs) about adolescent idiopathic scoliosis (AIS) and submitted them to ChatGPT 3.5, ChatGPT 4.0, and Google Bard [40]. The authors found that among all LLMs, responses were rated as “excellent” in 26% of cases, and the most advanced model tested, ChatGPT 4.0, led with 39% “excellent” ratings [40]. Raters further found that of the answers rated worse than “excellent”, 22% cited clear mistakes and 9% were off topic, highlighting the need for continuous improvement in empathy, contextual understanding, and accuracy before widespread clinical integration [40]. It’s important to note that since this study, more advanced LLMs such as ChatGPT 5.0 and Gemini have emerged, and offer expanded multimodal processing, longer context handling, and refined reasoning abilities that could address several of the limitations identified in these earlier models [42,43]. Thus, as these models continue to mature, it isn’t unreasonable to say that LLMs could become essential tools for translating complex medical language into clear, accessible information that supports shared decision-making.

Collectively, advances in hybrid modeling, NLP, and LLMs signal a shift toward integrative AI systems capable of synthesizing heterogeneous data sources and contextual information that more closely reflect real-world clinical complexity. Rather than optimizing performance within isolated data silos, these approaches aim to bridge imaging, clinical documentation, patient-reported experience, and biomechanical reasoning within unified analytic frameworks. However, their translational readiness remains constrained by familiar challenges outlined in previous sections. Hybrid models often rely on specialized imaging protocols or institution-specific data that limit transportability, while NLP and LLM-based systems are highly sensitive to documentation practices, linguistic variability, and data provenance. Moreover, as model architectures grow increasingly complex and generative, ensuring interpretability, factual reliability, and appropriate clinical oversight becomes essential to prevent automation bias or uncritical adoption. Without addressing these concerns, we are concerned that clinicians may over-trust an AI output simply because it came from an algorithm, even if it conflicts with clinical judgment, contextual knowledge, or patient-specific factors.

## 7. Author’s Perspective: Has the 2019 Vision Been Achieved?

At the close of 2019, Galbusera et al. recognized AI and ML as emerging forces in spine research [1]. They highlighted how these technologies demonstrated promising accuracy in diagnostic and imaging applications. They also emphasized, however, that the widespread implementation of these models was limited by the lack of large databases and the ability for many AI predictions to be properly generalized. Looking ahead, the authors believed that the potential impact of accurate and reliable automated diagnostic tools would be “enormous” [1]. They expected that AI’s use would continue to increase and that significant advances in imaging analysis, outcome prediction, and clinical decision support systems would result. The authors stressed, however, that realizing this potential was contingent upon the development of large, high-quality datasets, as well as open access to ML models and training data to foster public trust, improve accountability, and reduce prediction bias [1].

Five years later, AI continues to display enormous promise in revolutionizing the practice of spine surgery. The initial success in imaging analysis has rapidly evolved, moving beyond basic segmentation, where high accuracy appears to now be routine, to achieving diagnostic parity with expert human performance [15,18]. Such advancement has also fueled the development of models capable of generating high-fidelity synthetic images and multimodal data integration [22,23]. Concurrently, predictive modeling has transitioned from mostly descriptive analyses towards delivering individualized forecasting of functional outcomes and quality-of-life scores [11,28]. These sophisticated models are able to provide objective risk stratification, thereby addressing the historic problem of highly discordant, surgeon-based risk estimates [33]. Efforts to ensure model interpretability in order to overcome the “black box” nature of AI decision making have also been made but continue to require significant progress [11].

The scope of AI has even expanded into new frontiers within the past five years. In the pre-2019 era, machine learning applications were dominated by supervised learning techniques, but the exploration of unsupervised techniques has enabled the identification of hidden clusters of datapoints that may possess prognostic value that isn’t immediately obvious to researchers. As the volume and complexity of medical data continue to expand, strong unsupervised learning algorithms will become increasingly essential. Imaging, genomics, wearable sensors, electronic health records, and open access databases generate massive volumes of data that require sophisticated analytical approaches, and advances in large-scale data acquisition through NLP and LLMs are also proving to be increasingly valuable [4,39,41].

It must be acknowledged, however, that while these technologies are rapidly evolving and often demonstrate impressive quantitative performance, performance metrics such as high Dice similarity coefficients, strong accuracy percentages, and high intersection-over-union scores can be misleading in isolation. Technical success does not guarantee clinical readiness or ethical generalizability and this concern, first underscored by Galbusera et al. in 2019, remains highly relevant today [1]. Many contemporary AI models perform well on internal datasets yet fail when tested against external or heterogeneous data sources. In a systematic review of 83 published studies reporting 86 DL algorithms for image-based radiologic diagnosis, Yu et al. analyzed the performance of these algorithms when applied to external validation datasets [44]. The authors found that the vast majority of algorithms demonstrated diminished performance on the external dataset compared with internal performance [44].

It is therefore imperative that spine surgeons and researchers interpret performance metrics with caution and place greater emphasis on whether a model can achieve true generalizability [45]. This, however, also requires some clarification. As spine researchers, it is paramount that we remain vigilant in how we interpret the terms “validation” and “generalizability”. Oftentimes, models with great potential to inform patients and support medical decisions fail to reach clinical implementation due to “lack of external validation” being cited as the reason [45]. In a commentary by la Roi-Teeuw et al., the authors provide a valuable perspective on this issue, arguing that external validation, while critical, is neither a universal guarantee of model readiness nor a one-time procedural step [45]. The authors note that many clinical prediction models fail to progress to implementation because their validation and deployment strategies are misaligned with their intended clinical context [45]. This observation directly parallels the current trajectory of AI in spine research, where models often exhibit high internal performance metrics but limited real-world implementation [26,44].

Per la Roi-Teeuw et al., there are three misconceptions to be aware of that clarify this gap between validation and implementation [45]. The first is the belief that external validation inherently provides stronger evidence of performance than internal validation. The authors argue that the two should not be viewed as hierarchically superior to one another but rather as serving complementary purposes [45]. Internal validation focuses on performance within the model’s development population, while external validation focuses on transportability of model performance to a new setting [45]. The authors emphasize that validation results are context-specific and must correspond to the model’s intended use [45]. A local model designed for one specific hospital may only require internal validation, as its intended use does not require transportability. Poor external validation results from another hospital could lead to an unwarranted loss of confidence in the model’s true clinical utility [45].

To strengthen generalizability in spine AI, the authors recommend several strategies that can readily translate to the domain [45]. These include aligning validation design with the intended scope of model use, conducting multiple targeted external validations across relevant patient populations and imaging systems, addressing data representativeness gaps that hinder transportability, and continuously monitoring model performance and recalibrating as clinical environments evolve [45]. Importantly, these steps reframe generalizability as a dynamic, iterative process rather than a static achievement [45]. In doing so, they remind researchers and clinicians that validation is not a procedural hurdle to clear, but an ongoing responsibility to ensure that AI-driven tools remain consistently reliable, interpretable, and equitable across the diverse realities of clinical spine care.

## 8. Conclusions

Applications for AI in spine research exist across several areas, including imaging analysis, risk prediction, qualitative phenotyping, hybrid modeling and LLM-driven NLP. Since 2019, AI in spine research has matured from proof-of-concept experiments to matching or even surpassing expert performance in several of these areas. Persistent challenges in data quality, heterogeneity, interpretability, and context-appropriate validation still remain. Surgeons should be cautious when observing high internal performance metrics of AI models, as these alone cannot guarantee safe, equitable, or clinically meaningful deployment. The future of AI in spine research appears to be extremely promising, and we believe that continued progress will require sustained investment in creating large multicenter datasets, context-aligned validation strategies, and close collaboration between data scientists, spine surgeons, and regulatory bodies.

## Data Availability

Although no data was reported in this study, the search strategies used can be made available from the corresponding author on request.

## Supplementary Materials

The following supporting information can be downloaded at: https://www.mdpi.com/article/10.3390/jcm15020820/s1. Supplementary Table S1. Definitions and Representative Applications of Machine Learning (ML) in Spine Research. Supplementary Table S2. Overview of commonly used model types in spine research. Supplementary Table S3. Common performance metrics cited in AI and ML research.

## Abbreviations

The following abbreviations are used in this manuscript:

AI	Artificial intelligence
ML	Machine learning
DL	Deep learning
NLP	Natural language processing
LLM	Large language model
ANN	Artificial neural network
CNN	Convolutional neural network
RNN	Recurrent neural network
VLD-Net	Vertebrae localization and detection network
CART	Classification and regression tree
RF	Random forest
SVM	Support vector machine
PCA	Principal component analysis
Q	Query
K	Key
V	Value
GAN	Generative adversarial network
TP	True positive
TN	True negative
FP	False positive
FN	False negative
AUC-ROC	Area under the curve-Receiver operator characteristic
AUC-PR	Area under the curve-Precision recall
MRI	Magnetic resonance imaging
IoU	Intersection over union
LSS	Lumbar spinal stenosis
MC	Modic changes
MAIR	Multimodal articulated image registration
Syn-STIR	Synthetic short tau inversion recovery
Acq-STIR	Acquired short tau inversion recovery
MSTFI	Metastatic spinal tumor frailty index
CCI	Charlson comorbidity index
mFI-5	Modified 5-item frailty index
GNRI	Geriatric nutritional risk index
HFRS	Hospital frailty risk score
OR	Odds ratio
CI	Confidence interval
ASD	Adult spinal deformity
PRO	Patient-reported outcomes
OFC	Old/frail/content
OFD	Old/frail/distressed
ORC	Old/resilient/content
YRC	Young/resilient/content
HR	Hazard ratio
EOS	Early-onset scoliosis
DCM	Degenerative cervical myelopathy
cMRI	Community magnetic resonance imaging
aMRI	Advanced magnetic resonance imaging
EHR	Electronic health record
CSRS	Cervical Spine Research Society
VADER	Valence-aware dictionary sentiment reasoner
MAUDE	Manufacturer and user facility device experience
FAQ	Frequently asked questions
AIS	Adolescent idiopathic scoliosis
