# Supplementary material for: Advent of Artificial Intelligence in Spine Research: An Updated Perspective
Source: J Clin Med. 2026 Jan 20;15(2):820. doi: 10.3390/jcm15020820 (PMC12841851; doi:10.3390/jcm15020820)
Supplement: Supplementary file 1 [file jcm-15-00820-s001.zip › jcm-4070168-supplementary.pdf]

Supplementary Table S1. Definitions and Representative Applications of Machine Learning (ML) in Spine Research.

Type of ML	Description of technique	Applications in spine research
Supervised learning	Model learns from labeled data. Each input has a known output, such as a diagnosis or surgical outcome. Algorithms iteratively adjust parameters to minimize prediction error.	Automated Pfirrmann grading of lumbar disc degeneration from MRI [1].
Unsupervised learning	Model receives unlabeled data and identifies inherent structure or clusters without predefined categories.	k-means clustering of adult spinal deformity patients into prognostic phenotypes [2].
Semi-supervised learning	Model starts by learning patterns from a small, labeled dataset and then applies those learned patterns to assign "pseudo-labels" to the unlabeled data. It treats then treats those pseudo-labeled examples as additional training material, improving its ability to generalize without needing massive manual annotation.	Semi-supervised hybrid spine network (SSHSNet) for simultaneous segmentation of vertebral bodies and intervertebral discs in 3D magnetic resonance images [3].
Self-supervised learning	Model learns directly from unlabeled data by generating its own pseudo-labels through pretext tasks that reveal underlying structure or relationships within the data. It then uses these self-generated labels to train itself, improving feature representation and performance without any human annotation.	Segmentation of intervertebral discs, while simultaneously learning to ignore variations in image intensity caused by different MRI scanners to improve generalizability [4].
Reinforcement learning	Model learns by interacting with an environment and receiving feedback ("rewards" or "penalties") to maximize performance over time.	Localization of vertebrae on X-rays and estimation of Cobb angles using the Vertebrae Landmark Detection Network (VLD-Net) [5].

Supplementary Table S2. Overview of commonly used model types in spine research.

Model type	Learning mechanism	Operational framework
Linear regression	Supervised learning	Linear regression models how an outcome is expected to change on average with changes in one or more input features. It assumes a linear relationship between the predictors and the outcome and attempts to fit a straight line through the data. During training, the model computes the difference between its predictions and the true values, and adjusts the coefficients so that the overall error, typically measured by least squares, is minimized. Linear regression works best when the true relationship between inputs and the outcome can be reasonably approximated by a straight line [6–8].
Logistic regression	Supervised learning	Logistic regression models how the probability of a categorical outcome changes with one or more input features. Instead of predicting a continuous value, it estimates the likelihood of belonging to a particular binary class by modeling the log-odds of the outcome as a linear combination of the predictors. This linear expression is then passed through a sigmoid function, which converts the result into a probability between 0 and 1. During training, the model compares its predicted probabilities to the true labels and adjusts its coefficients to minimize a loss function such as cross-entropy. Because it relies on this linear relationship in the log-odds space, logistic regression works best when the boundary separating the classes can be reasonably approximated by a straight line or hyperplane [9,10].
Decision tree (CART)	Supervised learning	Decision trees classify or predict outcomes by repeatedly splitting the data into smaller, more uniform groups based on feature values. At each step, the algorithm selects the feature and threshold that best separates the data. These splits create a branching, flowchart-like structure in which each internal node represents a decision rule and each terminal node represents a final prediction. Because the model partitions the feature space into simple, interpretable regions, decision trees work well when relationships in the data can be captured by a series of rule-based splits rather than a single linear trend [11,12].
Random forest (RF)	Supervised learning	Random forests build on the idea of decision trees by creating many of them, each trained on a slightly different subset of the data and features. During training, every tree learns its own series of splits, producing a collection of diverse decision rules rather than relying on a single model. For prediction, the forest combines the outputs of all trees, either through majority vote for classification tasks or via averaging for regression tasks. This helps smooth out the noise and biases of any one tree. The ensemble approach improves accuracy and substantially reduces overfitting, making random forests especially useful when the underlying patterns are complex and cannot be captured by a single, deep tree [13].

Support vector machine (SVM)	Supervised learning	Support vector machines classify data by finding the line or hyperplane that best separates the classes in the feature space. Rather than simply drawing any boundary, the algorithm searches for the boundary that maximizes the distance between the boundary and the closest data points from each class. When data is not linearly separable, SVMs can apply kernel functions to project the inputs into a higher-dimensional space where a separating hyperplane can be found. Because of this emphasis on margin maximization and flexible boundary shapes, SVMs work well when class differences are subtle but can be revealed through an appropriately chosen feature transformation [14].
k-means clustering	Unsupervised learning	k-means clustering organizes unlabeled data into groups by identifying collections of points that share similar characteristics. The algorithm begins by choosing a set of cluster centers and then repeatedly assigns each data point to the nearest center based on its Euclidean distance. After assignment, the centers are updated to reflect the average position of the points within each group, and this process continues until the cluster structure stabilizes. Because k-means relies on this iterative process of geometric similarity, it works best when the underlying clusters are relatively compact and well separated in the feature space [15].
Principal component analysis (PCA)	Unsupervised learning	Principal component analysis reduces the dimensionality of complex datasets by identifying the directions, or principal components, along which the data vary the most. The algorithm computes these components by finding orthogonal axes that capture the greatest amount of variance, allowing the data to be represented in fewer dimensions with minimal loss of information. During this process, each data point is projected onto the new axes, creating a transformed space that highlights the most informative patterns in the data. Because PCA preserves the dominant sources of variation while discarding noise and redundancy, it is especially useful for improving interpretability, visualization, and downstream modeling [16].
Convolutional neural network (CNN)	Supervised learning	Convolutional neural networks are deep learning models designed to learn directly from image data by detecting spatial patterns that appear across different locations within the image. They use convolutional filters, which are small, trainable “squares” that slide across the input, to capture features such as edges, textures, or shapes that are important for tasks like identifying structures on MRI scans or X-rays. As the network progresses through multiple layers, it builds increasingly abstract representations of the image, allowing it to recognize complex anatomical patterns. Because CNNs exploit this hierarchical feature extraction, they are especially powerful for medical imaging applications where subtle structural differences carry diagnostic significance [17].
Recurrent neural network (RNN)	Supervised learning	Recurrent neural networks are deep learning models designed to handle temporal or sequential data by incorporating feedback connections that allow information to persist across time steps. Unlike feedforward

		networks, which process each input independently, RNNs maintain a hidden state that captures what the model has learned from previous inputs, enabling it to recognize patterns that unfold over time. This makes RNNs especially well-suited for longitudinal clinical data, sequential measurements, or any setting where past information influences future outcomes. Because they explicitly model temporal dependencies, RNNs are useful when the relationships within the data cannot be fully understood from isolated observations alone [18].
Transformer architecture	Supervised learning	Transformers replace the step-by-step processing of RNNs with a self-attention mechanism that allows the model to evaluate all tokens in a sequence simultaneously. After each token is converted into an embedding, the model applies three learned linear transformations to generate query (Q), key (K), and value (V) vectors. The Q and K vectors determine how strongly tokens should attend to one another by computing dot-product similarity scores, which function like testing whether one token's "key" fits another's "keyhole." These scores are normalized into attention weights that specify which other tokens provide the most relevant contextual information. The model then uses these weights to compute a weighted sum of the V vectors, producing a new context-enriched representation of each token. Multi-head attention repeats this process in parallel, allowing the transformer to capture multiple types of relationships simultaneously. This combination of parallel processing and attention-based contextualization is what gives transformers their exceptional performance across language, imaging, and biomedical domains [19].
Autoencoder	Self-supervised learning	Autoencoders are neural networks that learn compact, information-rich representations of data by training the model to reconstruct its own input. They consist of an encoder, which compresses the data into a lower-dimensional latent space, and a decoder, which attempts to rebuild the original input from this compressed representation. Because the model must preserve the most essential features to achieve accurate reconstruction, autoencoders naturally learn efficient structure within the data. This makes them especially useful for tasks such as denoising, dimensionality reduction, anomaly detection, or serving as a feature extractor for downstream models [20].
Generative adversarial network (GAN)	Self-supervised learning	Generative adversarial networks consist of two neural networks: a generator, which creates synthetic data, and a discriminator, which evaluates whether that data is real or artificial. These networks are trained together in a competitive, "adversarial" setup. The generator progressively learns to produce outputs that are increasingly realistic, while the discriminator becomes better at identifying subtle differences between real and generated samples. Through this dynamic interplay, GANs learn to model complex data distributions and can generate highly realistic synthetic images, such as MRI scans, that support data augmentation, modality translation, or simulation studies in medical research [21].

Ensemble	Supervised learning	Ensemble models improve predictive performance by combining multiple individual models, each capturing different aspects of the data. Two of the most widely used ensemble approaches in biomedical and clinical prediction are Random Forests (discussed earlier) and boosted tree models such as Gradient Boosting Machines, XGBoost, and LightGBM. Boosted tree models train trees sequentially so that each one corrects the errors made by the previous one. By pooling the strengths of many weaker learners, ensemble methods substantially reduce variance, mitigate overfitting, and produce more robust and accurate predictions than any single model alone [22].
Hybrid	*Supervised learning	Hybrid models combine multiple sources of information and often multiple model architectures to produce more comprehensive and clinically meaningful predictions. These approaches can integrate heterogeneous data types, such as imaging, clinical variables, biomechanics, and sensor-derived metrics, and process them through different specialized components. Such component designs include pairing a CNN for image feature extraction with a rule-based model for structured data. By leveraging the strengths of each modality and architecture, hybrid systems capture complementary patterns that would be missed by any single model alone, making them particularly effective in complex clinical decision-support settings [23].

*Although hybrid or multimodal models combine multiple architectures and incorporate diverse data types, the underlying learning process is typically supervised. This is because the final model still optimizes its parameters using labeled clinical or imaging data, even if the internal architecture is more complex or integrative.

Supplementary Table S3. Common performance metrics cited in AI and ML research.

Metric	Description
Accuracy	*Proportion of all predictions that are correct. Calculated as (TP + TN) / (TP + FN + FP + TN). Can be misleading when classes are imbalanced because it treats all errors equally. Commonly used when evaluating baseline performance in balanced classification tasks [24].
Sensitivity	Measures the model's ability to correctly identify positive cases. Calculated as TP / (TP + FN). High sensitivity reduces false negatives. Commonly used when the model must minimize false negatives, such as detecting disease or pathology [24].
Specificity	Measures the model's ability to correctly identify negative cases. Calculated as TN / (TN + FP). High specificity reduces false positives. Commonly used when the model must minimize false positives, such as ruling out disease or avoiding unnecessary interventions [24].
Positive Predictive Value	Proportion of predicted positives that are true positives. Calculated as TP / (TP + FP). High precision means the model rarely reports false positives. Commonly used when assessing how reliable positive model outputs are in clinical decision-making [24].
Negative Predictive Value	Proportion of predicted negatives that are true negatives. Calculated as TN / (TN + FN). Indicates reliability of negative predictions. Commonly used when evaluating confidence in negative findings, especially for screening or exclusion tasks [24].
F1 Score	Harmonic mean of precision and recall. Balances false positives and false negatives. Useful when neither precision nor recall alone captures performance. Commonly used when evaluating models built on imbalanced datasets, such as rare disease detection [24].
AUC-ROC	†Quantifies how well a model separates positive from negative cases across every possible decision threshold. The ROC curve plots the model's sensitivity versus its false positive rate at all thresholds, and the AUC represents the probability that the model will assign a higher score to a randomly chosen positive case than to a randomly chosen negative case. An AUC of 0.5 indicates no discriminative ability (equivalent to chance), whereas an AUC of 1.0 reflects perfect discrimination. Commonly used when comparing overall classification performance across all possible decision thresholds [24].
‡AUC-PR	Summarizes a model's performance using precision and recall across all possible decision thresholds. Unlike the ROC curve, which plots sensitivity against false positive rate, the precision–recall curve focuses on how many of the model's positive predictions are actually correct (precision) and how many true positives it successfully identifies (recall). Higher AUC-PR values indicate that the model maintains strong precision without sacrificing recall, even when true positives are uncommon. Commonly used when evaluating models on highly imbalanced datasets, where ROC curves may overestimate performance [25].
Cohen's Kappa	Measures agreement between two raters or methods while accounting for agreement due to random chance. Values range from -1 to 1, with higher values indicating stronger true agreement. Commonly used when assessing agreement between an AI model and one or more human experts while adjusting for chance [24,26].
Lin's Concordance Correlation Coefficient	Measures how closely two continuous or ordinal numerical ratings agree by assessing both correlation and accuracy. Unlike Pearson's correlation, which only evaluates the strength of a linear relationship, CCC penalizes systematic bias or scale differences between methods. Values range from -1 to 1, with higher values indicating stronger true

	agreement. Commonly used when evaluating agreement on severity grading scales that are treated as numeric (e.g. Pfirrmann grades, stenosis severity scores) [27].
Mean Absolute Error	Average magnitude of errors without regard to direction. Provides an intuitive measure of typical prediction error in real units (e.g. degrees, mm, etc.). Commonly used when evaluating typical prediction error in continuous regression tasks [28].
Root Mean Squared Error	Square root of the average squared error. Penalizes large errors more heavily than MAE. Sensitive to outliers. Commonly used when trying to penalize large errors more heavily in regression models [28].
§R^2	Represents the proportion of variance in the dependent variable explained by the model. Values range from 0-1, where higher values indicate that the model captures more of the variability in the data. Commonly used when trying to explain how well a regression model explains variance in the outcome [24,29].
Dice Similarity Coefficient	Quantifies how much the predicted and true segmentation masks overlap, by comparing the amount of overlap relative to the average size of the two masks. ||Defined as $2\|A∩B\| / (\|A\| + \|B\|)$, with values from 0 (indicating no overlap) to 1 (indicating perfect overlap). Commonly used when evaluating segmentation performance in medical imaging (e.g. disc, vertebrae, tumor boundaries) [24,30].
Intersection-over-Union	Quantifies how much the predicted and true segmentation masks overlap by comparing the amount of overlap to the size of their combined region. ||Defined as $\|A∩B\| / \|A∪B\|$, with values from 0 (indicating no overlap) to 1 (indicating perfect overlap). IoU is a more stringent measurement compared to Dice, because it penalizes boundary and size errors more. Commonly used in similar use cases to Dice, but in cases where boundary accuracy really matters [24,30].
Brier Score	Measures accuracy of probabilistic predictions by computing mean squared difference between predicted probabilities and actual outcomes. Lower values = better calibration. Commonly used when evaluating calibration of probabilistic predictions, such as risk prediction models [29].

*TP = True Positive; TN = True Negative; FN = False Negative; FP = False Positive; †AUC = area under the curve; ROC = receiver operator characteristic; ‡PR = precision-recall; §Note: R^2 reflects only how well the model fits the overall trend. It does not indicate how accurate the predictions are, nor does it assess calibration or the magnitude of individual errors. ||A = predicted mask, which represents all pixels the model thinks are part of the structure; B = ground truth mask, which represents all pixels the human expert labels as part of the structure.

References:

1. Jamaludin, A., Kadir, T. & Zisserman, A. SpineNet: Automated classification and evidence visualization in spinal MRIs. *Med Image Anal* **41**, 63–73 (2017).
2. Mohanty, S. *et al.* Machine learning clustering of adult spinal deformity patients identifies four prognostic phenotypes: a multicenter prospective cohort analysis with single surgeon external validation. *Spine J* **24**, 1095–1108 (2024).
3. Huang, J. *et al.* Spine Explorer: a deep learning based fully automated program for efficient and reliable quantifications of the vertebrae and discs on sagittal lumbar spine MR images. *Spine J* **20**, 590–599 (2020).
4. Fiorentino, M. C. *et al.* An intensity-based self-supervised domain adaptation method for intervertebral disc segmentation in magnetic resonance imaging. *Int J Comput Assist Radiol Surg* **19**, 1753–1761 (2024).
5. Xiang, S. *et al.* VLD-Net: Localization and Detection of the Vertebrae From X-Ray Images by Reinforcement Learning With Adaptive Exploration Mechanism and Spine Anatomy Information. *IEEE J Biomed Health Inform* **29**, 4969–4980 (2025).
6. Lunt, M. Introduction to statistical modelling: linear regression. *Rheumatology (Oxford)* **54**, 1137–1140 (2015).
7. Roustaei, N. Application and interpretation of linear-regression analysis. *Med Hypothesis Discov Innov Ophthalmol* **13**, 151–159 (2024).
8. Sebastião, Y. V. & St Peter, S. D. An overview of commonly used statistical methods in clinical research. *Semin Pediatr Surg* **27**, 367–374 (2018).
9. Schober, P. & Vetter, T. R. Logistic Regression in Medical Research. *Anesth Analg* **132**, 365–366 (2021).
10. Bewick, V., Cheek, L. & Ball, J. Statistics review 14: Logistic regression. *Crit Care* **9**, 112–118 (2005).
11. Blockeel, H., Devos, L., Frénay, B., Nanfack, G. & Nijssen, S. Decision trees: from efficient prediction to responsible AI. *Front. Artif. Intell.* **6**, 1124553 (2023).
12. Elhazmi, A. *et al.* Machine learning decision tree algorithm role for predicting mortality in critically ill adult COVID-19 patients admitted to the ICU. *J Infect Public Health* **15**, 826–834 (2022).
13. Breiman, L. Random Forests. *Machine Learning* **45**, 5–32 (2001).
14. Rodríguez-Pérez, R. & Bajorath, J. Evolution of Support Vector Machine and Regression Modeling in Chemoinformatics and Drug Discovery. *J Comput Aided Mol Des* **36**, 355–362 (2022).

15. Ikotun, A. M., Ezugwu, A. E., Abualigah, L., Abuhaija, B. & Heming, J. K-means clustering algorithms: A comprehensive review, variants analysis, and advances in the era of big data. *Information Sciences* **622**, 178–210 (2023).
16. Jolliffe, I. T. *Principal Component Analysis*. (Springer-Verlag, New York, 2002). doi:10.1007/b98835.
17. Yamashita, R., Nishio, M., Do, R. K. G. & Togashi, K. Convolutional neural networks: an overview and application in radiology. *Insights Imaging* **9**, 611–629 (2018).
18. Mienye, I. D., Swart, T. G. & Obaido, G. Recurrent Neural Networks: A Comprehensive Review of Architectures, Variants, and Applications. *Information* **15**, 517 (2024).
19. Vaswani, A. *et al.* Attention Is All You Need. Preprint at https://doi.org/10.48550/ARXIV.1706.03762 (2017).
20. Chen, S. & Guo, W. Auto-Encoders in Deep Learning—A Review with New Perspectives. *Mathematics* **11**, 1777 (2023).
21. Koshino, K. *et al.* Narrative review of generative adversarial networks in medical and molecular imaging. *Ann Transl Med* **9**, 821 (2021).
22. Kern, C., Klausch, T. & Kreuter, F. Tree-based Machine Learning Methods for Survey Research. *Surv Res Methods* **13**, 73–93 (2019).
23. Kline, A. *et al.* Multimodal machine learning in precision health: A scoping review. *npj Digit. Med.* **5**, 171 (2022).
24. Powers, D. M. W. Evaluation: from precision, recall and F-measure to ROC, informedness, markedness and correlation. https://doi.org/10.48550/ARXIV.2010.16061 (2020) doi:10.48550/ARXIV.2010.16061.
25. Saito, T. & Rehmsmeier, M. The precision-recall plot is more informative than the ROC plot when evaluating binary classifiers on imbalanced datasets. *PLoS One* **10**, e0118432 (2015).
26. McHugh, M. L. Interrater reliability: the kappa statistic. *Biochem Med (Zagreb)* **22**, 276–282 (2012).
27. Lin, L. I. A concordance correlation coefficient to evaluate reproducibility. *Biometrics* **45**, 255–268 (1989).
28. Willmott, C. J. & Matsuura, K. Advantages of the mean absolute error (MAE) over the root mean square error (RMSE) in assessing average model performance. *Climate Research* **30**, 79–82 (2005).
29. Steyerberg, E. W. *et al.* Assessing the performance of prediction models: a framework for traditional and novel measures. *Epidemiology* **21**, 128–138 (2010).

30. Taha, A. A. & Hanbury, A. Metrics for evaluating 3D medical image segmentation: analysis, selection, and tool. *BMC Med Imaging* **15**, 29 (2015).
